# Supplementary material for: Adoption of evidence-based medicine: A comparative study of hospital and community pharmacists in Saudi Arabia
Source: PLoS One. 2025 May 23;20(5):e0324620. doi: 10.1371/journal.pone.0324620 (PMC12101653; doi:10.1371/journal.pone.0324620)
Supplement: Attachment — Submitted filename: Response to Reviwers.docx [file pone.0324620.s002.docx]

January 17, 2025

Dear Editor and Reviewers,

We are pleased to resubmit our manuscript titled “Adoption of Evidence-Based Medicine: A Comparative Study of Hospital and Community Pharmacists in Saudi Arabia‎” for consideration as an original article. Our co-authors for this manuscript are Fahad Alzahrani, Nawaf Almutairi, Abdullah Aloufi1, Abdulmalik kattan, Abdulaziz Hakeem, Mohammed Alharbi, Naif Alarawi, Haifa Abdulrahman Fadil1, and Ehsan Habeeb1.

We are grateful for the time and effort that you and the reviewers have dedicated to evaluating our manuscript (PONE-D-24-39283 ). We have carefully incorporated your suggestions and made revisions accordingly. The revised ‎manuscript now provides a more comprehensive and robust framework for interpreting the ‎results and drawing meaningful conclusions.‎, which were highlighted using the tracking change function in the manuscript.

Please find our responses below, highlighted in blue, along with a point-by-point response ‎to each of the reviewers’ comments. We hope that you find the revisions satisfactory. Should you have any ‎further suggestions or require any additional information, please do not hesitate to contact us. ‎We are more than willing to address any remaining concerns.‎

Thank you for your consideration. We look forward to the opportunity to share our findings with the readership.

Sincerely,

Fahad Alzahrani, BPharm, RPh, MSc, DIPBA, PhD, C-KPI, CSPP

Associate Professor, Behavioral and Administrative Pharmacy

Department of Pharmacy Practice, College of Pharmacy,

Taibah University, Madinah, Saudi Arabia

Tel: +966561130077

Email: fzahrani@taibahu.edu.sa

Editor general comments:

i. **Comment:** Introduction: Please ensure that your introduction is scientifically sound. Begin with a general overview and then narrow down to specific details. Be sure to incorporate existing solutions to the problem, highlight the research gap, and reference previous studies.

**Response:** Thank you for your valuable feedback. We have thoroughly revised the introduction to make sure of scientific soundness and proper scholarly progression. **Comment:** Language Editing: I recommend enhancing the overall English language quality of your manuscript.

**Response:** Thank you for your valuable comment. We have carefully revised the manuscript to enhance its clarity, precision, and academic tone. Here are the specific improvements made.

ii. **Comment:** Abstract: Please revise your abstract to ensure it is more compelling and meets the journal’s standards.

**Response:** Thank you for your feedback. We have thoroughly revised the abstract to make it more compelling and aligned with journal standards.

**Reviewer's Responses**

**Reviewer #1 Comments:** Actuality, evidence-based medicine has become an essential discipline for the decision-making process in the individual care of patients, with judicious and conscious use of the best scientific evidence available.
The relevance of this study takes root in the need to incorporate in an accurate way the basic knowledge of EBM into daily pharmacist’s practice. There are still few studies that evaluate the knowledge, attitudes, and practices of pharmacists regarding the practice of EBM.

**Responses:** We appreciate your insights and have strengthened our manuscript to emphasize these important points better. First, we enhanced the introduction to establish the study's significance better. Second, we have strengthened the justification for the research gap.

**Reviewer #2 comment:** The study employed convenience sampling and a combination of purposive and snowball sampling methods to select research subjects, which might lead to selection bias among respondents. Additionally, only 38.1% of the recruited samples were ultimately included in the analysis, thereby limiting the validity and representativeness of the results. Therefore, it is recommended to supplement the analysis with a comparative study of some of the unincluded samples further to clarify the authenticity and representativeness of the results.

**Response:** We appreciate your concerns about the sampling methodology. This study used two sampling techniques: convenience and snowball sampling. We acknowledge that our initial manuscript incorrectly included purposive samplingand have removed this reference. The text in the manuscript has been updated.

Regarding selection bias although convenience sampling can introduce selection bias, our choice was informed by both practical considerations and methodological precedent in clinical and social settings.[1-3]. We have addressed this limitation by explicitly acknowledging it in our limitations section.

Concerning the suggestion to conduct a comparative analysis with unincluded samples, we must note that the majority of excluded responses were due to incomplete data, specifically where participants discontinued upon reaching questions about their educational qualifications.

**References**

1.	Stratton SJ. Population research: convenience sampling strategies. Prehospital and disaster Medicine. 2021;36(4):373-4.

2.	Simkus J. Convenience sampling: Definition, method and examples. Retrieved Oktober. 2022;6:2022.

3.	Etikan I, Musa SA, Alkassim RS. Comparison of convenience sampling and purposive sampling. American journal of theoretical and applied statistics. 2016;5(1):1-4.
