# Supplementary material for: Adoption of evidence-based medicine: A comparative study of hospital and community pharmacists in Saudi Arabia
Source: PLoS One. 2025 May 23;20(5):e0324620. doi: 10.1371/journal.pone.0324620 (PMC12101653; doi:10.1371/journal.pone.0324620)
Supplement: Attachment — Submitted filename: Response to Reviwers (2).docx [file pone.0324620.s003.docx]

April 22, 2025

Dear Ms. Chaudhary,

We are pleased to resubmit our revised manuscript titled “Adoption of Evidence-Based Medicine: A Comparative Study of Hospital and Community Pharmacists in Saudi Arabia” for further consideration as an original article in *PLOS ONE*.

We would like to thank you and the reviewers once again for your constructive feedback. In this second revision, we have carefully addressed all remaining comments and made the necessary improvements. All changes are highlighted using the Track Changes feature.

A detailed, point-by-point response to the reviewers’ comments (with responses highlighted in blue).

We believe these updates enhance the clarity and strength of our work. Should you require any additional information, we would be happy to provide it.

Thank you for your continued consideration. We look forward to your response.

Sincerely,

Fahad Alzahrani, BPharm, RPh, MSc, DIPBA, PhD, C-KPI, CSPP

Head of Statistical Consultation and Survey Research Unit

Associate Professor, Behavioral and Administrative Pharmacy

Department of Pharmacy Practice, College of Pharmacy,

Taibah University, Madinah, Saudi Arabia

Tel: +966561130077

Email: fzahrani@taibahu.edu.sa

**Reviewer's Responses**

**Reviewer #2**

1. **Comments:** The response rate after sampling in this study was only 38.15%, and the average age of the participants was relatively young (31 years old), so the representativeness of the research results is limited and there may be selective bias. It is suggested to supplement the overall number of research subjects that meet the inclusion and exclusion criteria, as well as compare the basic information of the sample obtained from this sampling with the overall population.

**Responses:** Thank you for this valuable comment. We acknowledge that the response rate of 38.15% and the relatively young average age of participants may limit the representativeness of our findings and introduce potential selection bias. We have addressed this limitation in the revised manuscript under the "Study Limitations" section.

Regarding the suggestion to supplement the number of research subjects and compare the basic characteristics of the sample with the overall population, we appreciate the recommendation. However, due to the design of our online survey using Google Forms' branching logic, we automatically excluded pharmacy technicians holding diploma degrees, a vital segment of the healthcare workforce in Saudi Arabia, along with individuals who declined participation or submitted incomplete surveys. The survey system immediately terminated responses for diploma holders after the education level question, and incomplete surveys were not stored in our database. Similarly, we have no demographic or comparative data for non-respondents. Therefore, while we recognize this as a significant limitation, we are unable to perform the suggested comparison between respondents and non-respondents or analyze potential differences between complete and incomplete survey responses. We have highlighted this limitation and recommended that future research use stratified sampling and larger, more inclusive datasets to enhance representativeness and allow for more detailed comparisons.

2. **Comment:** In addition, the research topic is a comparison between hospital and community pharmacists, and there is a lack of corresponding KAP comparison data in the results. Suggest adding baseline data comparison between hospitals and community pharmacists; Comparison of KAP survey results between hospital and community pharmacists.

**Response:** Thank you for your constructive feedback. We have addressed your concerns as follows:

1. Baseline Data Comparison:

We have expanded Table 1 to include detailed baseline demographic characteristics comparing hospital and community pharmacists.

2. KAP Survey Results Comparison:
   We have enhanced our results section, which provides a detailed comparison of KAP scores between hospital and community pharmacists as follows:
   a. Knowledge Domain:
      Hospital pharmacists demonstrated significantly higher knowledge scores (M = 3.84, SD = 0.52) compared to community pharmacists (M = 3.70, SD = 0.57), with statistical significance (p = 0.03).
   b. Attitude Domain:
      No significant difference was observed in attitude scores between hospital pharmacists (M = 3.83, SD = 0.50) and community pharmacists (M = 3.76, SD = 0.53; p = 0.60).
   c. Practice Domain:
      Hospital pharmacists showed significantly higher practice scores (M = 3.89, SD = 0.51) compared to community pharmacists (M = 3.76, SD = 0.58; p = 0.02).

**Reviewer #3**

**Clarity and Consistency in Writing**

1. **Comment:** The manuscript is generally well-written, but some sections contain redundancy. For instance, in the introduction, the definition of EBM is repeated in multiple ways. Consider merging these statements for conciseness.

**Response:** Thank you for your comment. We have revised the introduction to be more concise by removing redundant definitions of EBM and streamlining the content.

2. **Comment:** Some sentences are overly long and complex, making them harder to follow. For example, in the introduction, "It combines the best available scientific evidence with the clinician's expertise and the patient's values to guide medical decisions effectively." This could be revised for better flow: "EBM integrates scientific evidence, clinical expertise, and patient values to improve medical decision-making."

**Response:** Thank you for your feedback. We have revised the sentence as suggested.

Research Justification and Novelty

3. **Comment:** The study’s importance is well-stated, but there is limited emphasis on the novelty of the research. Consider explicitly stating how this study fills a gap in previous research. For example:
   a. Does this study provide the first comparative analysis of hospital vs. community pharmacists in Saudi Arabia?
   b. How does it add to previous research findings on EBM adoption in pharmacy practice?
      Strengthening this section will help establish the study's significance.

**Response:** We appreciate your feedback. Thank you for your input. We have revised our manuscript to emphasize the significance of our study, particularly highlighting its status as the first comparative analysis of EBM adoption between hospitals and community pharmacists in Saudi Arabia, in both the concluding paragraph of the introduction and the opening section of the discussion. In addition, we have revised the discussion section to illustrate what our study adds to previous research.

Methodology

4. **Comment:** The response rate (38.15%) is relatively low. Was there any effort to mitigate potential non-response bias?

**Response:** Thank you for your valuable comment. While our response rate of 38.15% is lower than ideal, it aligns with similar pharmacy practice surveys conducted in Saudi Arabia. For instance, Al-Jazairi and Alharbi reported a 20% response rate among hospital pharmacists when assessing evidence-based practice [1]. Another cross-sectional study targeting hospital pharmacists yielded a response rate of 14.8% [2]. Additionally, the “National Survey of Drug Information Centers Practice in Saudi Arabia” reported a 40% response rate, involving pharmacists in drug information centers, which may include community pharmacists [3]. This limitation has been explicitly addressed in the revised manuscript, highlighting its potential implications for the generalizability and interpretation of the study findings.

5. Comment: The use of convenience and snowball sampling raises concerns about selection bias. While this is acknowledged, a stronger justification is needed to explain why this method was chosen over random sampling.

**Response:** While random sampling would be ideal, convenience and snowball sampling were employed due to several context-specific challenges. First, there was no comprehensive, accessible sampling frame of pharmacists in Saudi Arabia. For instance, Fathelrahman conducted a nationwide cross-sectional study assessing medical device-related counseling practices among community pharmacists in Saudi Arabia [4]. They employed convenience sampling due to the lack of a national registry or contact database for pharmacists, which prevented the use of probability sampling methods and stratification by geographical region. This limitation underscores the challenges in obtaining a representative sample of pharmacists in specific regions like the Madinah region of Saudi Arabia. Consequently, our study utilized convenience and snowball sampling techniques to reach pharmacists across diverse practice settings, particularly community pharmacists who are typically harder to access through institutional channels. While this approach may introduce selection bias, it aligns with established practices in pharmacy research where complete listings of the target population are unavailable. The Study Sampling and Response Rate section has been updated to reflect this rationale.

6. **Comment:** The exclusion criteria are mentioned briefly. Were there any specific reasons for excluding pharmacists from the pharmaceutical industry or academia? Clarifying this will strengthen the study's rigor.

**Response:** Thank you for your observation. Our study specifically focused on hospital and community pharmacists, as they are the primary healthcare professionals actively involved in patient care and clinical decision-making informed by evidence-based medicine (EBM). This choice is supported by prior research indicating that these pharmacists are key users of EBM in daily practice, particularly in medication therapy management, clinical interventions, and patient-centered care [5, 6]. Pharmacists working in industry and academia were excluded, as they typically do not hold active patient care licenses and are not routinely engaged in clinical decision-making that necessitates EBM application. This exclusion aligns with the methodology of similar studies investigating EBM use among frontline healthcare providers, which have similarly omitted participants from non-clinical sectors [7-9]. By targeting licensed pharmacists involved in direct patient care, our study offers a more accurate reflection of EBM implementation in routine pharmacy practice. We have clarified this rationale in the revised *Study Population* subsection of the Methods section to enhance transparency and methodological rigor.

**Statistical Analysis - Additional Clarifications**

7. **Comment:** The manuscript states that Bloom's threshold was used to categorize knowledge, attitude, and practice scores. While this is a reasonable approach, it would be beneficial to justify why this method was chosen over other classification methods.

**Response:** Thank you for this thoughtful comment**.** The use of Bloom's threshold in categorizing knowledge, attitude, and practice (KAP) scores is a common methodological approach in cross-sectional studies, as demonstrated by multiple sources, particularly in the absence of standardized benchmarks [10-13]. While none of the provided studies explicitly justify why Bloom’s cut-off was selected over other classification methods, their consistent application of this method supports its validity as a standardized tool in KAP research. We have added a brief justification in the Methods section to clarify the rationale for this choice.

**Comment:** The use of net agreement and net frequency scores is well-documented, but some readers may not be familiar with these terms. Consider adding a brief explanation or reference in the Methods section.

**Response:** Thank you for your helpful comment. We have added a short explanation and supporting reference in the Methods section to define these measures and their relevance in KAP studies.

8. **Comment:** The effect size (Cohen’s d = 0.15 - 0.16) is interpreted as "small." This should be explicitly stated in the Results section, as some readers may not be familiar with effect size thresholds.

**Response:** Thank you for the comment. Cohen’s d is a commonly used measure of effect size that indicates the standardized difference between two means. According to Cohen’s (1988) guidelines, an effect size of 0.2 is considered small, 0.5 is medium, and 0.8 or above is large. A small effect (d ≈ 0.2) suggests a modest difference between groups that, while statistically significant, may have limited practical significance [14]. We have revised the Methods and Results sections to clearly state that the observed effect sizes fall within the small range.

**Interpretation of Findings**

9. **Comment:** The results indicate that pharmacists with less than one year of experience had higher EBM practice scores than more experienced pharmacists. This is an interesting finding but needs further exploration. Could it be that recent graduates receive better EBM training? If so, how can this be addressed in continuing education programs?

**Response:** Thank you for this thoughtful and insightful comment. We agree that the finding warrants further exploration. One possible explanation is that recent graduates may have received more formal and updated training in evidence-based medicine (EBM) as part of evolving pharmacy curricula [14-16]. In contrast, pharmacists with more years of experience may not have had the same level of EBM emphasis during their initial training, which may explain the lower practice scores among this group. This trend is consistent with findings in other health professions, where integrated EBM curricula have been shown to significantly improve information literacy and evidence application skills among students and recent graduates [17]. This highlights a potential gap in ongoing professional development and underscores the importance of integrating targeted EBM content into continuing education and professional development programs. We have expanded the Discussion section to include this interpretation and its implications.

10. **Comment:** Community pharmacists had lower EBM scores than hospital pharmacists. The manuscript attributes this to differences in professional settings, but other factors (e.g., lack of access to resources, workload differences) should be discussed in more depth.

**Response:** Thank you for your comment. We do agree that the observed differences in EBM scores between community and hospital pharmacists may be influenced by a broader range of factors beyond the practice setting alone. Community pharmacists often face barriers such as limited access to clinical resources, lack of institutional support, and higher workload and time constraints, which may hinder their ability to engage with EBM regularly. In contrast, hospital pharmacists may have better access to multidisciplinary teams, structured protocols, and clinical decision-support tools. We have expanded the Discussion section to reflect these considerations and provide a more comprehensive interpretation of the findings.

11. **Comment:** The misconception that EBM ignores clinical experience was reported by 41.7% of participants. This is a crucial barrier and should be highlighted in the discussion as a key area for intervention.

**Response:** Thank you for your observation. We have emphasized this point in the revised Discussion section and identified it as a key area for educational and institutional intervention.

**Practical Implications and Recommendations**

12. **Comment:** The manuscript does a good job of discussing barriers to EBM adoption, but the recommendations for overcoming these barriers are somewhat vague.
    a. What specific training strategies should be implemented?
    b. Would workshops, online courses, or mentorship programs be more effective?
    c. How can institutions support pharmacists in overcoming time constraints?

**Response:** We appreciate your comment. We have expanded the Discussion section to include evidence-based strategies for overcoming key barriers to EBM adoption. For training, structured continuing education programs that combine interactive workshops, case-based learning, and evidence retrieval exercises are recommended. Online courses and webinars offer flexibility and broad accessibility, especially for community pharmacists. Mentorship programs led by experienced clinical pharmacists can also provide personalized guidance and reinforce practical application. To address time constraints, institutions can support EBM practice by integrating clinical decision support tools into pharmacy workflows, allocating protected time for professional development, and fostering a culture that values evidence-based care. These enhancements have been added to the revised manuscript to provide a more comprehensive roadmap for improving EBM uptake.

**Minor Changes**
**Abstract:**

13. **Comment:** EBM in Saudi Arabia’s pharmacies is an under-researched area despite its importance in pharmacy practice.” Consider rewording to: "Despite its importance in pharmacy practice, EBM adoption in Saudi Arabian pharmacies remains under-researched."

**Response:** Thank you for the suggestion. The sentence has been updated to: *“Despite its importance in pharmacy practice, EBM adoption in Saudi Arabian pharmacies remains under-researched.”*

**References**

1.	Al-Jazairi AS, Alharbi R. Assessment of evidence-based practice among hospital pharmacists in Saudi Arabia: attitude, awareness, and practice. International Journal of Clinical Pharmacy. 2017;39:712-21.

2.	Ajabnoor AM, Cooper RJ. Pharmacists’ prescribing in Saudi Arabia: cross-sectional study describing current practices and future perspectives. Pharmacy. 2020;8(3):160.

3.	Alomi Y, Alghamdi S, Alattyh R. National Survey of Drug Information Centers Practice in Saudi Arabia: Drug Monitoring and Patient Counseling at Ministry of Health Hospitals. J Pharmacovigil. 2017;5(246):2.

4.	Fathelrahman AI. Medical Devices-Related Counseling Practices Among Community Pharmacists: A Nationwide Cross-Sectional Study from Saudi Arabia. Integrated Pharmacy Research and Practice. 2021:113-25.

5.	Lee KMK, Koeper I, Johnson ME, Page A, Rowett D, Johnson J. Multidisciplinary perspectives on roles of hospital pharmacists in tertiary settings: a qualitative study. International Journal for Quality in Health Care. 2024;36(1):mzad110.

6.	Toklu HZ. Promoting evidence-based practice in pharmacies. Integrated Pharmacy Research and Practice. 2015:127-31.

7.	Lüthold RV, Cateau D, Jenkinson SP, Streit S, Jungo KT. Pharmacists’ attitudes towards interprofessional collaboration to optimise medication use in older patients in Switzerland: a survey study. BMC health services research. 2024;24(1):849.

8.	Khan N, McGarry K, Naqvi AA, Iqbal MS, Haider Z. Pharmacists' viewpoint towards their professional role in healthcare system: a survey of hospital settings of Pakistan. BMC health services research. 2020;20:1-15.

9.	Alexa J, Bertsche T. An online cross-sectional survey of community pharmacists to assess information needs for evidence-based self-medication counselling. International Journal of Clinical Pharmacy. 2023;45(6):1452-63.

10.	Wang L, Abualfoul M, Oduor H, Acharya P, Cui M, Murray A, et al. A cross-sectional study of knowledge, attitude, and practice toward COVID-19 in solid organ transplant recipients at a transplant center in the United States. Frontiers in Public Health. 2022;10:880774.

11.	Alibrahim D, El Mahalli A. The knowledge, attitude and practice level of dental auxiliaries regarding oral health care for pregnant patients in the eastern province of Saudi Arabia. F1000Research. 2023;11:216.

12.	Deeb N, Naja F, Nasreddine L, Kharroubi S, Darwiche N, Hwalla N. Nutrition knowledge, attitudes, and lifestyle practices that may Lead to breast Cancer risk reduction among female university students in Lebanon. Nutrients. 2024;16(7):1095.

13.	Baig I, Azam S. Knowledge Level and Practice of Primary School Teachers Regarding Traumatic Dental Injuries and Their Emergency Management in Twin Cities. Journal of Health and Rehabilitation Research. 2024;4(2):1617-22.

14.	Ramis M-A, Chang A, Nissen L. Strategies for teaching evidence-based practice to undergraduate health students: a systematic review protocol. JBI Evidence Synthesis. 2015;13(2):12-25.

15.	Chandran V, Thunga G, Pai G, Khan S. 26 Application and retention of evidence based practice skills: students and practitioner’s perspectives from an indian healthcare institution. Royal Society of Medicine; 2018.

16.	Poirier TI. A new vision for pharmacy education: It is time to shift the old paradigm and move forward. American Journal of Pharmaceutical Education. 2007;71(5):103.

17.	Wang C, Yao Y, Chen Y, Chen J. The impact of evidence-based medicine curricula on information literacy among clinical medical undergraduates and postgraduates in China. BMC Medical Education. 2025;25(1):520.
